# Supplementary material for: Relationship between maltreatment and mental health in adolescents: A school-based study in Indonesia
Source: PLoS One. 2024 Nov 12;19(11):e0310533. doi: 10.1371/journal.pone.0310533 (PMC11556743; doi:10.1371/journal.pone.0310533)
Supplement: S1 Table — (DOCX) [file pone.0310533.s001.docx]

**S1 Table. Validity and Reliability of the instruments**

| Psychometric attribute | Instruments | |
| --- | --- | --- |
|  | ICAST | SDQ |
| Outfit mean square  Mean  Standard deviation | 1.09  0.44 | 1.04  0.17 |
| Reliability | 0.99 | 1 |
| Item separation | 11.83 | 19.05 |
| Cronbach’s alpha | 0.8 | 0.7 |
