# Supplementary material for: Relationship between maltreatment and mental health in adolescents: A school-based study in Indonesia
Source: PLoS One. 2024 Nov 12;19(11):e0310533. doi: 10.1371/journal.pone.0310533 (PMC11556743; doi:10.1371/journal.pone.0310533)
Supplement: Attachment — Submitted filename: Response to Reviewers.Cover Letter PlosOne_July 2024.docx [file pone.0310533.s002.docx]

21^th^ July 2024

Dear Editorial Team at PLOS ONE,

We are very grateful for the reviews provided by the editors and each of the external reviewers of this manuscript. The comments are encouraging and the reviewers appear to share our judgement that this study and its results are important.

Please find a point-by-point response to the issues raised by the reviewer. Newly added words and sentences are highlighted by using track change word in revised manuscript version and in yellow text highlight color in this response letter.

We hope we can respond to the issue raised and submit a revised version of the manuscript for consideration for “"Relationship between Maltreatment and Mental Health in Adolescents: A School-based Study from Indonesia"

Sincerely,

Prof. DR. Meita Dhamayanti, MD

Department of Child Health

Hasan Sadikin General Hospital

Faculty of Medicine Universitas Padjadjaran

Bandung, Indonesia

Manuscript ID: PONE-D-23-24880R1

# Title of paper:

Relationship between Maltreatment and Mental Health in Adolescent: A School Based Study from Indonesia

**Response to Reviewer 1 Comments**

We wish to thank the Reviewer 1 for his/her excellent comments and suggestions. We have responded to each of the issues raised. All changes are highlighted by using track change word in revised manuscript version and in yellow text highlight colour in this response letter

**Reviewer #2:**

**Point 1**: Thank you for carrying out some of the suggested changes. However, I could not find the point where the authors state the study hypotheses.

**Response 1:** Thank you very much for your comment. We carefully considered your suggestion and added the hypothesis in INTRODUCTION, line 75

This study hypothesis that physical and psychological maltreatment correlates with mental health problem in adolescent.

**Point 2**: Moreover the discussion about participation and information bias (as well as the study design) should be corroborated with at least one more sentence briefly explaining in which way the results of this study might be affected by these bias. Once addressed these points I am happy to endorse the submission

**Response 2:** Thank you very much for your comment.

the results of this study might be affected by these bias, the statement has also added in DISSCUSION (line 205 -210) as follow:

Also, the separation of categories in this study as the junior and high school level is analyzed in anticipation of bias resulted from an adolescence developmental phase. Since three psychosocial developmental phase of adolescence are early phase or middle school (10-14 years age) , middle phase or high school (15-18 years age) and late phase approximate college years, hence a trajectory between phase involve amount of change in cognitive, emotional, and social behavior [53]. The participation and information bias may lead to the limitation of this stud**y**.
