# Supplementary material for: Relationship between maltreatment and mental health in adolescents: A school-based study in Indonesia
Source: PLoS One. 2024 Nov 12;19(11):e0310533. doi: 10.1371/journal.pone.0310533 (PMC11556743; doi:10.1371/journal.pone.0310533)
Supplement: Attachment — Submitted filename: Response to the Editor 27 Augt 2024.docx [file pone.0310533.s003.docx]

27^th^ August 2024

Dear Miquel Vall-llosera Camps
Senior Staff Editor at PLOS ONE,

I am pleased to resubmit for your consideration a manuscript entitled **"Relationship between Maltreatment and Mental Health in Adolescents: A School-based Study from Indonesia"** for publication in PLOS ONE. This manuscript have revised the text and fixed the grammatical errors and improved the overall readability of the text by professional scientific editing service as your recommendation

Upon this resubmission , we provide:
* The EDITAGE professional service that edited our manuscript
* A copy of manuscript with tract changes by either highlighting them or using track changes (uploaded as a supporting information file)
* A clean copy of the edited manuscript (uploaded as the new manuscript file)

We believe that the manuscript aligns well with the scope and interests of PLOS ONE, and we are confident that it will make a valuable contribution to the existing literature on this subject matter. This submission represents original research that has not been previously published, and it is not under consideration for publication elsewhere. All co-authors have thoroughly reviewed and approved the content of this manuscript.

Thank you for considering our manuscript for publication in PLOS ONE. We appreciate the opportunity to share our work with the wider scientific community and contribute to the advancement of knowledge in this field. Should you require any further information or have any questions, please do not hesitate to contact me.

We look forward to your feedback and the possibility of publication in PLOS ONE.

Sincerely,

Prof. DR. Meita Dhamayanti, MD

Department of Child Health

Hasan Sadikin General Hospital

Faculty of Medicine Universitas Padjadjaran

Bandung, Indonesia
